# Trimeric heptad repeat synthetic peptides HR1 and HR2 efficiently inhibit HIV-1 entry

**DOI:** 10.1042/BSR20192196

**Published:** 2019-09-24

**Authors:** Olfa Mzoughi, Meritxell Teixido, Rémi Planès, Manutea Serrero, Ibtissem Hamimed, Esther Zurita, Miguel Moreno, Giovana Granados, Faouzi Lakhdar-Ghazal, Lbachir BenMohamed, Ernest Giralt, Elmostafa Bahraoui

**Affiliations:** 1 INSERM, U1043, CPTP, CHU purpan, Toulouse, France; 2 CNRS, U5282 CPTP, CHU purpan, Toulouse, France; 3 Université Paul Sabatier, CPTP, CHU purpan, Toulouse, France; 4 Institute for Research in Biomedicine (IRB Barcelona), The Barcelona Institute of Science and Technology, Baldiri Reixac, 10, Barcelona 08028, Spain; 5 Laboratory of Cellular and Molecular Immunology, Gavin Herbert Eye Institute, University of California Irvine, School of Medicine, Irvine, CA 92697, United States of America; 6 Department of Inorganic and Organic Chemistry, University of Barcelona, Martı i Franques 1-11, Barcelona 08028, Spain

**Keywords:** AIDS, C34, HIV-1, inhibitors, N36, trimers

## Abstract

The trimeric heptad repeat domains HR1 and HR2 of the human immunodeficiency virus 1 (HIV-1) gp41 play a key role in HIV-1-entry by membrane fusion. To develop efficient inhibitors against this step, the corresponding trimeric-N36 and C34 peptides were designed and synthesized. Analysis by circular dichroism of monomeric and trimeric N36 and C34 peptides showed their capacities to adopt α-helical structures and to establish physical interactions. At the virological level, while trimeric-C34 conserves the same high anti-fusion activity as monomeric-C34, trimerization of N36-peptide induced a significant increase, reaching 500-times higher in anti-fusion activity, against R5-tropic virus-mediated fusion. This result was associated with increased stability of the N36 trimer peptide with respect to the monomeric form, as demonstrated by the comparative kinetics of their antiviral activities during 6-day incubation in a physiological medium. Collectively, our findings demonstrate that while the trimerization of C34 peptide had no beneficial effect on its stability and antiviral activity, the trimerization of N36 peptide strengthened both stability and antiviral activity. This approach, promotes trimers as new promising HIV-1 inhibitors and point to future development aimed toward innovative peptide fusion inhibitors, microbicides or as immunogens.

## Introduction

Human immunodeficiency virus 1 (HIV-1) infects essentially CD4^+^ cells, including T-helper CD4 cells, monocytes, and macrophages [1–3]. Primo-infection is mediated by R5-tropic viruses that use CCR5 co-receptors. However, 8–10 years after post-infection, HIV-1 switches to a CXCR4 tropism in approximately 40–50% of HIV-1 infected patients [4,5]. In the absence of a Highly Active Antiretroviral Therapy (HAART), this switch is followed by a rapid progression of HIV-1-related acquired immune deficiency syndrome (AIDS) disease [6].

The life cycle of the HIV virus is mediated by a high affinity interaction of the external envelope glycoprotein gp120 in trimeric conformation with CD4 (*K*_d__=_ 10^−9^ M) which leads to conformational modifications in the structure of gp120, thus allowing its interaction with a subsequent co-receptor that can be either CCR5 or CXCR4 depending on viral tropism. Following interaction with its co-receptor, gp120 is shed, exposing the transmembrane protein gp41 ectodomain, a key factor of viral entry, as it undergoes a series of conformational changes to form at least five distinguishable and functional domains of the fusion-active state. These domains include: (***i***) the hydrophobic N-terminal fusogenic peptide, (***ii***) the peptide N36 derived from the N-terminal heptad repeat (NHRI), (***iii***) the linker region, (***iv***) the C34 peptide derived from the C-terminal heptad region (CHRII), and (***v***) the membrane proximal external region (MPER). Dynamically, following gp120–CD4–CCR5/CXCR4 interactions, the detachment of the gp120 makes the hydrophobic fusion peptide of gp41 accessible to insert into the lipid membrane of the target cell further allowing the interactions between three NHRI and CHRII domains to form a six-helix bundle complex [7–9]. This conformational change brings both the viral and cellular membranes into closer proximity to facilitate membrane fusion leading to viral entry into the host cell cytoplasm [10,11]. Thus, the interaction of NHRI–CHRII is a key step in the life cycle of HIV-1 and its blockade abolishes entry and thus viral replication.

A number of viruses of public health or economic interest use the equivalent of NHRI and CHRII to induce membrane fusion, including viruses from families of retroviridae (as HIV-1), orthomyxoviridae (influenza virus A) [12], filoviridae (Ebola virus) [13], paramyxoviridae (measles virus) [14] and coronaviridae (MERS and SARS viruses) [15]. Thus, understanding the underlying mechanisms of viral and cell membrane fusions at the molecular, structural and dynamic levels is an essential objective for the development of potent inhibitors of this crucial step. It is noteworthy to mention that achieving this goal goes well beyond applications for the treatment of HIV-1 as the majority of enveloped viruses use the mechanism of fusion between the viral and host membrane at either the cell surface or the endosomal level.

To date, a broad range of inhibitors targeting different key steps of the HIV-1 viral cycle, including reverse transcription (Nevirapine), processing of viral proteins (Saquinavir, Lopinavir), or integration of the provirus (Raltegravir), are available and used with success in the clinical treatment of HIV-1 infected patients. These treatments, named tritherapy or HAART are very efficient at controlling the viral load [16,17]. During the course of treatment, resistant viruses may emerge, however the antiretroviral arsenal currently available is sufficient to overcome this issue. However, a few days following HIV-1 transmission, a stable reservoir of CD4 T cells, latently infected with HIV-1, is established [18], constraining HIV-1 infected patients, even those harboring an undetectable viral load in the blood, to take these drugs for life [19]. Despite their effectiveness, all these anti-HIV-1 drugs act once the virus has entered target cells. Thus, it is very important to develop potent inhibitors that are able to block the virus before it enters the target cells and to block and/or limit the formation of the latent reservoir which is the main obstacle of HIV-1 cure. To date, two types of such inhibitors have been developed. The first type includes antagonists of the CCR5 co-receptor, represented by Maraviroc. This drug is used with great care in HIV-1 patients; it is delivered only in cases of failure of the usual tritherapy and, especially, in patients who harbor the R5 tropic virus only. Its administration to patients harboring X4-Tropic or X4/R5 dual-tropic viruses may facilitate the rapid selection of the more cytopathic X4-tropic viruses and thus the rapid progression to AIDS diseases [20]. The second type of HIV-1 entry inhibitors correspond to a different CHRII and, at a lower level, NHRI analog peptides that act by interfering with the formation of the NHRI–CHRII complex, thus inhibiting HIV-1 entry.

The first generation of fusion peptide analogs of CHRII, including T20, C34 and SJ-2179 related to HXB2 isolate sequence, blocks viral entry by competing with the interaction of CHRII with NHRI for the formation of the six-helix bundle [8,21–23]. The only fusion inhibitor peptide currently approved by the FDA for use in HIV-positive patients is T20, also named as DP178, Enfuvirtide or Fuzeon. T20 is a 36-residue peptide partly localized in the CHRII region (aa 638–673), inhibiting cell–cell fusion *in vitro* and HIV-1 replication of both CXCR4- and CCR5-tropic viruses at nanomolar levels [24,25]. However, the use of T20 has declined over the years because of its several limitations: it has a relatively short half-life (4 h) in plasma [21,26], large doses are required (90 mg twice daily) for its use, and it induces the emergence of resistant HIV-1 strains.

The second and third generations of fusion inhibitor peptides based on sequences from viral isolates different from HXBII, have been developed using analogs of NHRI and CHRII, including N36 [27], T-1249 [28], C34M2 [23], SC34EK peptides [29] Sifuvirtide [30], T-1144 [31] and T-2635 [31]. Despite their potent anti-HIV-1 activities *in vitro*, their use in HIV-1-infected patients remains limited by their low solubility and the lack of oral bioavailability. Unfortunately, none of these peptides have been approved by the FDA. Thus, there is a strong need to continue to improve HIV-1 entry inhibitors. Considering that gp120 and gp41 are present as trimers on the viral particles, the present study designed fusion inhibitor peptides based on trimeric forms of NHRI (N36) and CHRII (C34) peptides by using a minimal linker composed of five lysines. These trimers were shown to be stable in physiological-like medium containing human serum. These constructs are able to form stable complexes between trimeric NHRI and monomeric CHRII and complexes between trimeric CHRII and monomeric NHRI organized in α-helical structures. Furthermore, these two trimers are highly potent inhibitors of HIV-1-mediated membrane fusion, and block the replication of both HIV-1 R5-tropic and X4-tropic viruses. The present study contributes in providing trimers as promising novel HIV-1 inhibitors that may be used either as future innovative peptide fusion inhibitors or as immunogens.

## Materials and methods

### Trimers and peptide synthesis

Two trimeric peptides, trimer C34 and trimer N36, and two monomeric peptides, C34 and N36, were synthesized and used in the present study. The corresponding sequences used were derived from HIV-1 Lai and mimicked the N and C-terminal heptad repeats HR1 and HR2 domains (also named as N36 and C34, respectively). The N36 and C34 peptides were automatically synthesized in parallel on 330 mg of Rink amide-Gly-MBHA resin with low loading (0.3 mmol/g) on an Applied Biosystems 433A peptide synthesizer by using Fmoc chemistry. The MBHA resin HL (loading: 0.77 mmol/g, Novabiochem) was solvated by successive washing steps with DCM (CH_2_CL_2_) (3 × 1 min), 10% TFA in DCM (1 × 10 min), 1% DIEA (3 × 1min), DCM (3 × 1 min) and DMF (3 × 1 min). Fmoc-Gly-OH (0.1 mmol), pre-activated with TBTU/HOBt/DIEA (1:1:1.5) in DMF, was coupled. After 2 h, the resin was washed with DMF (3 × 1 min) and DCM (3 × 1 min) and the free amino groups were capped with acetic anhydride/DIEA/DMF (3:1:16) (2 × 30 min). The resin was washed with DMF (3 × 1 min) and the Fmoc group was removed with 50% piperidina/DMF (3 × 5 min). Rink amide linker was coupled after being pre-activated with TBTU/HOBt/DIEA (1:1:1.5) in DMF. Fmoc/tBu protected amino acids (Iris Biotech GmbH) were incorporated using standard procedures and TBTU/HOBt/DIEA as coupling agents. Fmoc group was removed by treatment with 22% piperidine and 0.07% Triton X-100 in DMF. Concomitant side chain deprotection and cleavage was performed by treatment with 5m l of a mixture of TFA/H_2_O/triisopropylsilane /ethanedithiol (92:4:2:2 v/v/v/v) at 0°C for 30 min and at room temperature for 1.5 h. TFA was removed by evaporation and the crude was precipitated with *tert*-butyl methyl ether. The trimers were synthesized using the same Fmoc chemistry, but manually. The products were characterized by HPLC, amino acid analysis and MALDI-TOF mass spectrometry.

### Circular dichroism analysis of peptides and trimers

The spectra were recorded on a JASCO J-810 spectropolarimeter with the CDF-426S Peltier unit using a quartz cuvette of 0.2 cm optical path length teflon stopper. The peptide was dissolved in sodium phosphate buffer pH 7.4. Solutions of 50, 25, 10 and 5 μM of peptides were prepared in aqueous buffer alone or in the presence of 30% of trifluoroethanol. The measurement was obtained between 190 and 260 nm with a data pitch of 0.2 nm, a band width of 1 nm and scanning speed of 0.8 nm/min for four accumulations. All spectra were corrected by subtracting the buffer spectrum. The circular dichroism (CD) data were expressed as molar ellipticity [θ] (deg.cm^2^.dmol^−1^) calculated by [θ_MR_] = 100θ/C_MR_ × l, where θ is the ellipticity in mdeg, C_MR_ is the molar concentration divided by the number of residues, and l is the path length of the cell in cm.

### Cell lines

HeLa-CD4-CCR5/CXCR4-LTR/β-gal cells, a gift from Dr. P. Charneau (Pasteur Institute, Paris, France), are HeLa cells stably expressing human CD4, human CCR5 and CXCR4 co-receptor (HeLa P4P5) and containing the LacZ gene under the control of the HIV-1 LTR promoter. HeLa-gp120/gp41LAI or HeLa-gp120/gp41ADA are HeLa cells stably expressing gp120/gp41 from HIV-1 LAI or HIV-1 ADA isolates. They were grown in complete Dulbecco’s modified Eagle medium in the presence of Geneticin (1 mg/ml) for HeLaCD4-LTR/β-gal or 1 μM of methotrexate for HeLa-gp120/gp41LAI and HeLa-gp120/gp41ADA.

### Syncytium formation

The synthesized monomeric and trimeric peptides were tested for their ability to inhibit syncytium at different concentrations (10^−6^–10^−11^ M). HeLa-CD4-CCR5-CXCR4 (2 × 10^4^ cells) were co-cultured with HeLa-gp120/gp41LAI or HeLa-gp120/gp41ADA (2 × 10^4^ cells) in 96-well plates in the presence of various concentrations of each monomeric or trimeric peptide. After 20 h, syncytia were scored by phase-contrast microscopy. The IC_50_, corresponds to the value of the peptide giving 50% of syncytia inhibition. This value is determined from the curves showing the % of inhibition of syncytia in function of the concentration of peptides used. The % of inhibition, at a given concentration, is calculated as follows: (number of syncytia in the presence of peptide/number of syncytia in the absence of peptide) × 100.

### Viruses

The R5 HIV-1 BaL and VN44 X4 were obtained from Dr. H. Hocini (ANRS, France). These viruses were amplified on HeLa P4P5 and the viral supernatant harvested at day 7 post-infection was titrated using the immunocapture p24 ELISA, aliquoted (100 ng of p24/ml) and stored at −80°C until use.

### β-gal assay

Cells were washed twice with PBS (0.5 mM MgCl_2_, 1 mM CaCl_2_), fixed with 0.5% glutaraldehyde for 10 min and washed twice with PBS. They were incubated for 3 h in a mixture (1 mg/ml X-Gal in PBS) containing potassium ferricyanide (5 mM), potassium ferrocyanide (5 mM) and MgCl_2_ (2 mM). The reaction was then stopped by removing the X-Gal reaction solution. Blue stained cells were scored under optical microscopy.

### Stability of monomeric and trimeric N36 and C34 peptides in the presence of human serum

To test the stability of monomeric and trimeric C34 and N36 peptides, each peptide (5 × 10^−7^ M) was incubated at 37°C for different times, 1–6 days, in cell culture medium complete with 50% of human serum AB and tested for the conservation of its capacity to inhibit syncytium formation.

## Results

### Design and synthesis of C34 and N36 trimers

**Figure 1:**
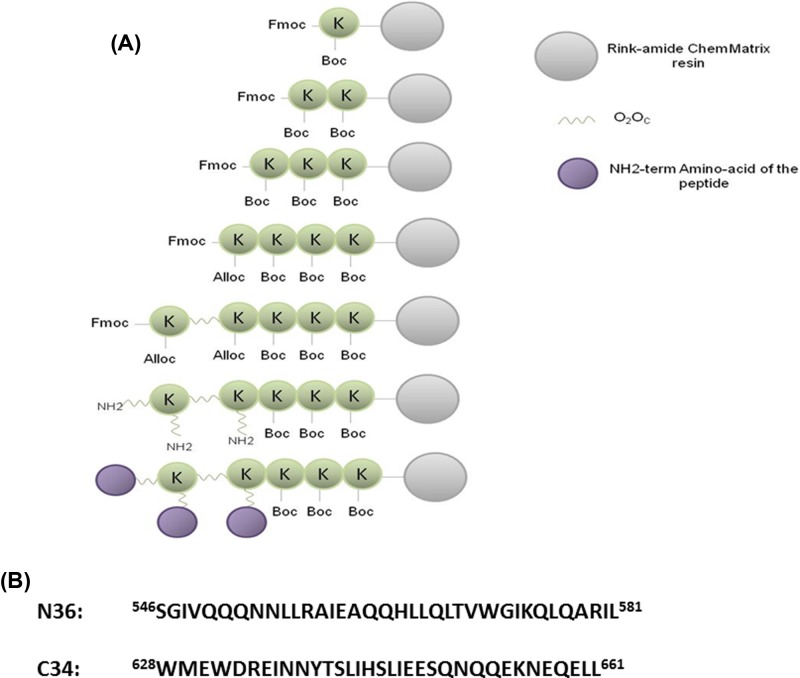
General strategy of the design and the synthesis of N36 and C34 trimeric peptides (**A**) Each trimer is composed of a linker that contains five lysines and four flexibility-enhancing spacer residues O_2_Oc at branching points and three copies of the same peptide: C34 or N36. (**B**) Primary sequences of N36 and C34 peptides corresponding to HR1 and HR2 domains, respectively, of HIV-1 Lai gp41.

C34 and N36 trimers were designed as previously reported [32,33] and synthesized manually as described [34,35]. Briefly, we first synthesized the linker on an Fmoc-rink-amide ChemMatrix resin modified to have a substitution of 0.2 mmol/g. The linker was composed of 5 lysines including four, 3.6 dioxaoctanoic acid (O_2_Oc) residues at branching points. The O_2_Oc spacer, placed between the branching lysines and the sequence of the N36 or C34 peptides, was introduced to improve the flexibility and the solubility of the trimeric peptides. Once the linker was synthesized, a small amount was cleaved and the mass of the linker was verified by mass spectrometry. After Fmoc deprotection, the peptides C34 or N36 were assembled on the linker. Since there are three free NH_2_ ends on the linker, three peptides were synthesized, linked simultaneously via each free NH_2_ of the lysine-linker (Figure 1).

In parallel, the corresponding monomeric C34 and N36 peptides were also synthesized using the same Fmoc-solid phase strategy. All the monomeric and trimeric peptides were purified by HPLC to at least 95% homogeneity. The homogeneity of each peptide was further confirmed by amino acid composition analysis and molecular mass determination by MALDI-TOF.

### CD analysis of the secondary structures and the capacity of interaction between monomeric and trimeric C34 and N36 peptides to form α-helix complexes

**Figure 2:**
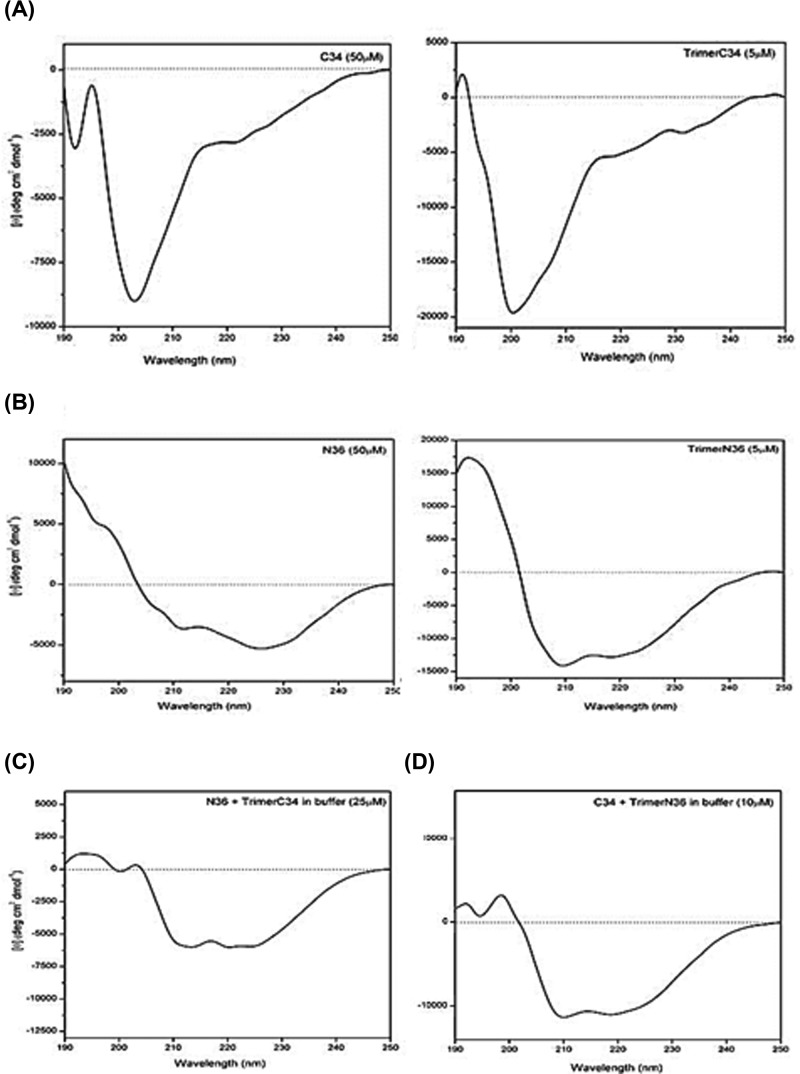
CD spectra of the various peptides and trimer–peptide complexes (**A**) CD spectra of C34 (left) and trimer C34 (right panel). (**B**) CD spectra of N36 (left panel) and trimer N36 (right). Peptides were used at 50 μM, and trimers at 5 μm in 10 mM sodium phosphate buffer pH 7.4, 50 mM potassium fluoride pH 7.4. (**C,D**) CD spectra of peptides in an equimolar mixture: monomer N36 + trimer C34 (C) and trimer N36 + monomer C34 (D). Spectra were obtained in aqueous solution. The apparition of a maximum at 192 nm and a double minimum at 208 and 222 nm were used as indicators of peptide interactions and of the formation of an α-helical structure.

Using CD approach, we studied the structure of C34 and N36 monomeric and trimeric peptides in aqueous solution. In the native-like structure of the HIV-1 transmembrane glycoprotein gp41, these peptides are folded into α-helical structures. Such structures seem to be absent from both the monomeric and the trimeric C34 peptides as shown by the absence in their spectra of the α-helical signatures presented by a maximum at 192 nm and a double minimum at 208 and 222 nm (Figure 2A). In contrast with C34 peptides, monomeric N36 and, even more so, the trimeric N36, showed a trend toward formation of α-helical structures (Figure 2B). This physical-spectral approach was also used to study the capacity of the interaction between equimolar concentrations of monomeric N36 and trimeric C34 (25 μM) and monomeric C34 and trimeric N36 (10 μM). Our results found a spectra with characteristics indicating the presence of α-helical structures with a maximum at 192 nm and double minimum at 208 and 222 nm (Figure 2C,D), demonstrating the direct interaction between monomeric N36 and trimeric C34 as well as between the monomeric C34 and trimeric N36 peptides leading to the formation of six helix-bundles, structures which play a crucial role in mediating the bringing together of viral and cell host membranes, thus catalyzing their fusion and allowing the virus to enter into the cytoplasm.

### Effects of monomeric and trimeric C34 and N36 peptides on HIV-1 gp120/gp41-mediated membrane cell–cell fusion

**Figure 3:**
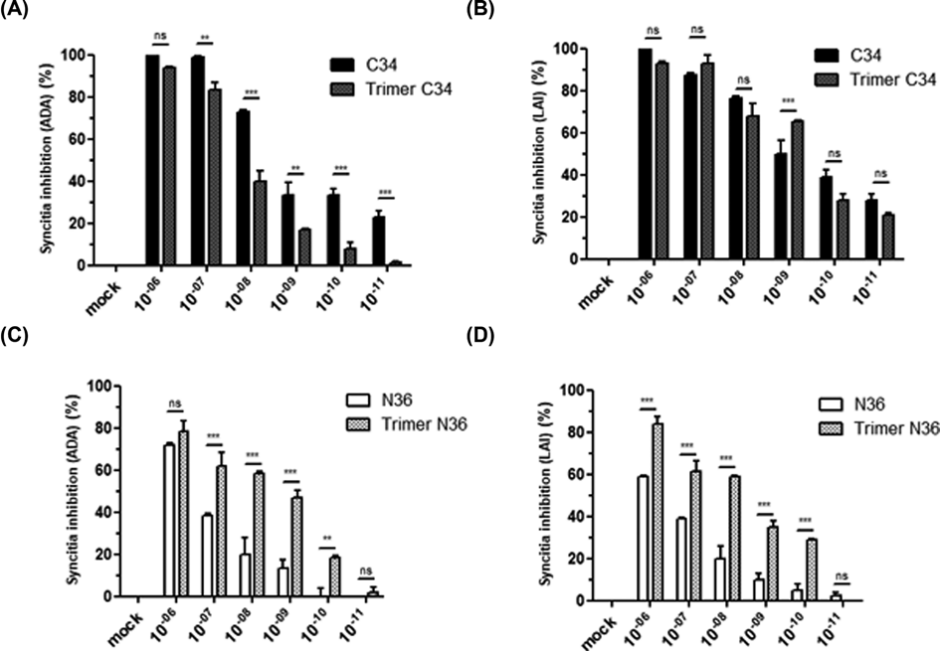
Characterization of the anti-fusion activity of C3 and N36 peptides HeLa cells expressing envelope glycoproteins from either HIV-1 ADA (R5-tropic virus) (**A,C**) or HIV-1 LAI (X4-tropic virus) (**B,D**) were incubated with HeLa cells expressing human CD4 receptor and CXCR4 or CCR5 HIV-1 co-receptors in the presence of escalating concentrations (10^−12^–10^−6^ M) of N36 or C34 peptides either in monomeric or trimeric conformation. After 20 h of incubation, syncytia formation was quantified by optical microscopy. The percentage of inhibition of syncytia formation was calculated. ‘mock’ corresponds to the syncytia formation obtained in the absence of peptide inhibitors but including the same medium as that used for the solubilization of the peptide inhibitor tested. Experiments were performed in triplicate and repeated three times. A representative experiment is shown as mean ± standard deviation. **, *P*<0.01; ***, *P*<0.0001

The antiviral activities of trimeric C34 and trimeric N36 peptides were examined in complementary assays, including inhibition of cell–cell fusion and inhibition of HIV-1 viral replication. In the first set of experiments, we tested the capacity of C34 and N36 peptides in either monomeric or trimeric forms to block HIV-1 entry. To this end, we used a co-culture assay of HeLa cells expressing envelope glycoproteins from HIV-1 LAI (X4-tropic virus) or HIV-1 ADA (R5-tropic virus) and HeLa cells expressing human CD4 receptor and CXCR4 or CCR5 HIV-1 co-receptors. Our results showed that both monomeric and trimeric C34 and N36 peptides were able to interfere with the formation of syncytia, but with different efficiencies (Figure 3). C34 peptide, in either the monomeric or trimeric form, inhibited more than 90% of syncytia formation by R5 or X4-tropic HIV-1 envelope glycoprotein at a concentration of 10^−7^ M. The trimeric structure of C34 peptide did not significantly improve its anti-fusion activity in comparison with the monomeric form, as both monomeric and trimeric peptides showed comparable IC_50_ values (in the nanomolar range) (Figure 3A,B).

In contrast with the monomeric C34 peptide, the monomeric N36 peptide exhibits a weaker anti-fusion activity with an IC_50_ value of 5 × 10^−7^ M for the inhibition of gp120_Ada_-dependent membrane fusion and an IC_50_ value of 5 × 10^−6^ M for the inhibition gp120_Lai_-dependent syncytia formation (Figure 3C,D). Interestingly, when the N36 peptide was tested in a trimeric structure, it became a more potent inhibitor of both gp120_Ada_ and gp120_Lai_-dependent syncytia formation with an IC_50_ of 10^−9^ and 5.10^−8^ M respectively. These results demonstrated that modification of N36 peptide from a monomeric to a trimeric structure enhanced its anti-fusion activity by approximately 500-times against R5-tropic and by 100-times against X4-tropic envelope glycoprotein-mediated fusions (Figure 3C,D).

**Figure 4:**
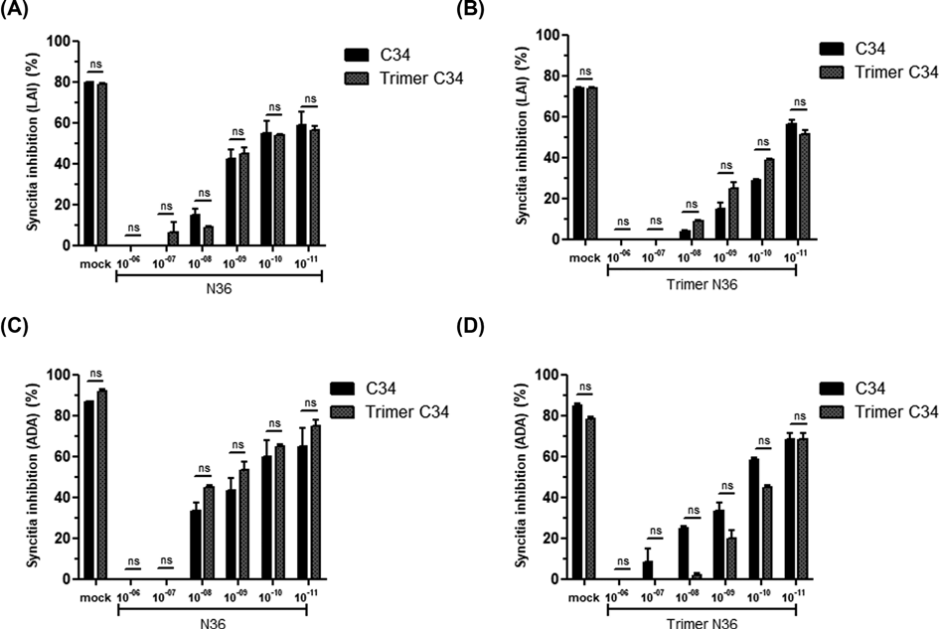
Anti-HIV-1 activities of monomeric and trimeric C34 and N36 peptides HeLa cells expressing envelope glycoproteins from either HIV-1 LAI (X4-tropic virus) (**A,B**) or HIV-1 ADA (R5-tropic virus) (**C,D**) were incubated with HeLa cells expressing human CD4 receptor and CXCR4 or CCR5 HIV-1 co-receptors in the presence of monomeric or trimeric C34 peptides used at 5 × 10^−8^ M that had previously been incubated with escalating amounts of monomeric or trimeric N36 peptides (10^−11^–10^−6^ M) and *vice versa*. After 20 h, the capacity of N36 peptide to cancel the C34 anti-fusion activity was evaluated. ‘mock’ corresponds to the syncytia formation obtained in the absence of peptide inhibitors but including the same medium as that used for the solubilization of the peptide inhibitor tested. Experiments were performed in triplicate and repeated three times. A representative experiment is shown as mean ± standard deviation. ns, nonsignificant.

At the molecular level, the inhibitory effect of N36 (HR1-analogs) and C34 (HR2-analogs) peptides is mediated by their capacities to bind to their cognate sequences on the HIV-1 gp41 envelope glycoproteins and thus to interfere with the formation of HR1–HR2 complexes, a crucial step in viral entry. We further characterized the molecular mechanism of C34 and N36 inhibitory fusion by using competition assay to test the effect of N36 peptide to cancel the inhibition of syncytia formation obtained by C34 peptide. To this end, monomeric and trimeric C34 peptides were incubated with escalating amounts of monomeric or trimeric N36 peptides (10^−11^–10^−6^ M). After 20 h, the capacity of the N36 peptide to cancel the C34 anti-fusion activity was evaluated. Our findings demonstrate that C34 peptides either in the monomeric or trimeric forms used at 5 × 10^−8^ M inhibit between 70 and 90% of syncytia formation between both HeLa-CD4-CCR5/HeLa-gp120/gp41_ADA_ and HeLa-CD4-CXCR4/HeLa-gp120/gp41_LAI_ (Figure 4A,B). The addition of escalating concentrations (10^−11^–10^−6^ M) of monomeric or trimeric N36 peptides induced a dose-dependent inhibition of C34 anti-fusion activity (Figure 4A,B). Syncytia formation between HeLa-CD4-CCR5/HeLa-gp120/gp41_ADA_ and HeLa-CD4-CXCR4/HeLa-gp120/gp41_LAI_ was completely restored in the presence of 10^−6^ and 10^−8^ M of monomeric or trimeric C34, respectively (Figure 4C,D). These results can be considered as an indirect demonstration of the capacity of monomeric and trimeric peptides to form stable complexes *in vitro*, not only with the HR1 or HR2 homologs present on the transmembrane gp41 envelope glycoproteins of HIV-1 Lai and ADA viral isolates expressed on HeLa Lai and HeLa ADA cells, but also with their soluble synthetic counterpart peptides in either monomeric or trimeric forms. In agreement with the best activity of trimeric N36 observed in the first test of fusion inhibition (Figure 3C,D), we also confirmed the better activity of trimeric N36 compared with monomeric N36. These results confirmed the importance of the relationship between the structure and the activity of N36 peptide (Figure 4). This structure relationship is, however, less important between monomeric and trimeric C34 peptides (Figure 3A,B).

**Figure 5:**
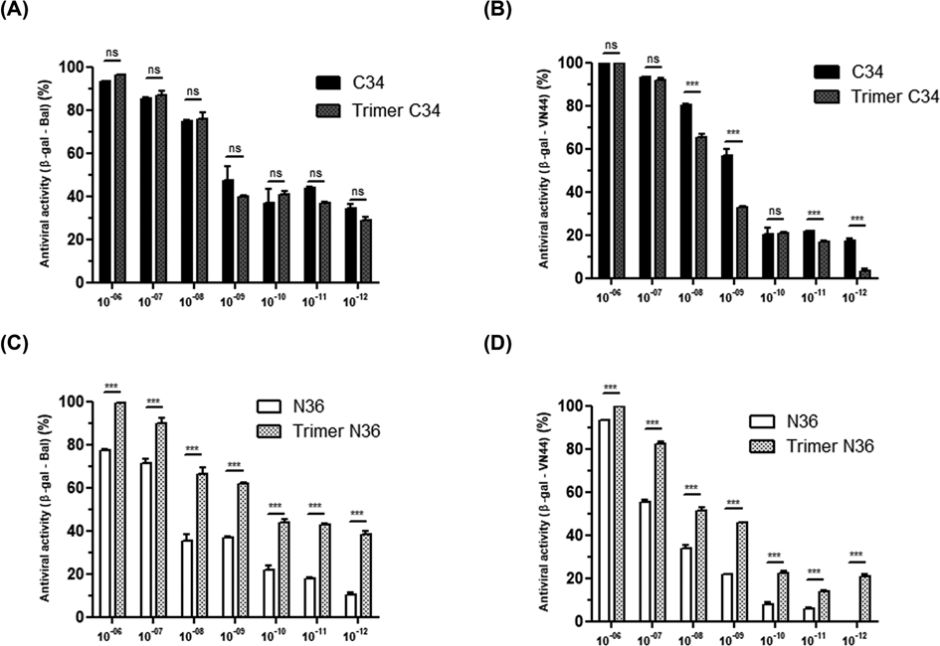
Anti-HIV-1 activity of C34 and N36 peptides and the trimer C34 and trimer N36 in a single infectivity assay HeLa CD4-CCR5/CXCR4-LTR/β-gal cells were infected for one viral cycle (20 h) with 0.1 ng of HIV-1 BaL (**A,C**) or VN44 (**B,D**). Viral replication was correlated directly with the transactivation of the Lac-Z gene by the early translated HIV-1 Tat gene. β-galactosidase activity was visualized by incubating cells in the presence of the substrate X-Gal which stained the cells blue after X-Gal degradation by the action of the LTR-driven expression of β-galactosidase. Experiments were performed in triplicate and repeated three times. A representative experiment is shown as mean ± standard deviation. *, *P*<0.05; **, *P*<0.001; ***, *P*<0.0001.

The antiviral activities of trimeric and monomeric C34 and N36 peptides were further characterized using two different viral replication assays. The first assay tested the antiviral effects of N36 and C34 peptides in a single viral cycle, while the second assay tested the antiviral activities of N36 and C34 peptides in multiple HIV-1 viral cycles. In the single viral cycle assay, monomeric and trimeric C34 and N36 peptides were tested at various concentrations (10^−6^–10^−10^ M) for their capacities to inhibit the infection of HeLa-CD4-CCR5/CXCR4-LTR-β*-gal* cells with the X4 tropic HIV-1-VN44 or the R5 tropic HIV-1-BaL isolates over a period of 24 h. This time lapse enabled the completion of the first steps of the viral cycle to be monitored, including adsorption, penetration and early genome expression. The assay is based on the ability of the early viral Tat protein to transactivate the expression of the *Lac-Z* gene, which has been placed under the control of HIV-1 LTR promotor. As in the syncytia inhibition assay, the antiviral activity was comparable between the monomeric and trimeric C34 peptides (Figure 5A,B), and a stronger antiviral activity was found with the trimeric N36 peptide, which inhibited the X4 tropic HIV-1 VN44 isolate with an IC_50_ of 10^−9^ M while the monomeric N36 peptide showed an IC_50_ of 5 × 10^−7^ M under the same conditions. These differences in antiviral efficiency between trimeric N36 and monomeric N36 were also found in their capacities to inhibit the R5 tropic HIV-1 Bal isolate with an IC_50_ of 5 × 10^−7^ and 5 × 10^−9^ M, respectively, in a similar assay. The ratio of IC_50_ values of monomeric N36 over those of trimeric N36 showed that trimeric N36 peptide was able to inhibit X4 tropic HIV-1 isolate and R5 tropic HIV-1Bal with an efficiency respectively 500- and 100-times better than the monomeric structure (Figure 5C,D).

**Figure 6:**
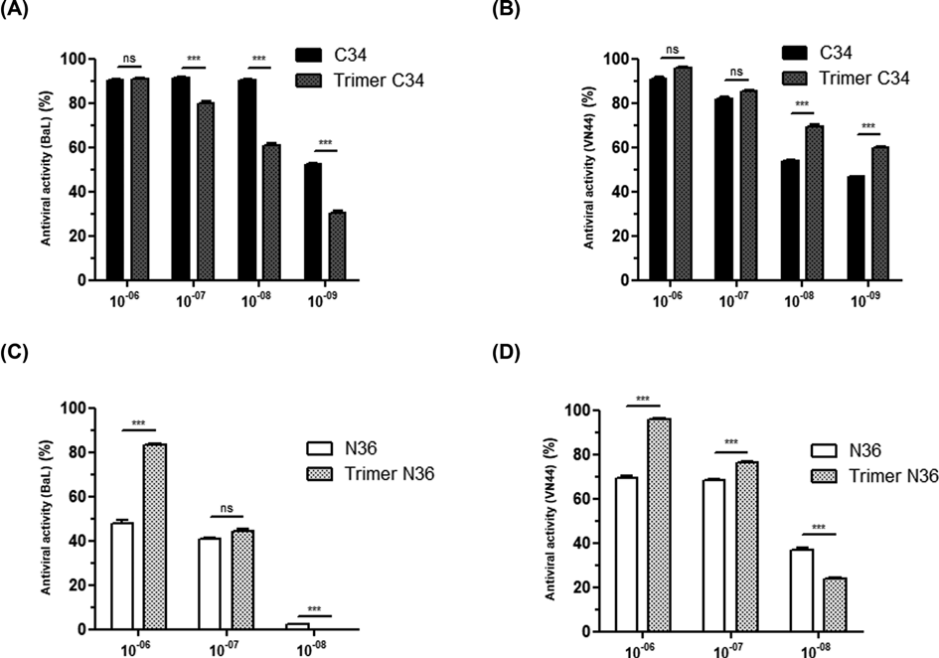
Effect of monomeric C34 and N36 peptides and the trimer C34 and trimer N36 on viral replication of X4 and R5 tropic viruses HeLa CD4-CCR5/CXCR4 were infected with 0.3 ng of the HIV R5 tropic BaL isolate (**A,C**) or the HIV X4 tropic VN44 isolate (**B,D**) viruses for 3 h. Three days post-infection, cell supernatants were collected and assayed by ELISA for p24 antigen. Uninfected cells treated in the same way were used as a control. Experiments were performed in triplicate and repeated three times. A representative experiment is shown as mean ± standard deviation. *, *P*<0.05; **, *P*<0.001; ***, *P*<0.0001; ns, non-significant.

In the second assay, the antiviral activity was evaluated by quantifying the amount of the HIV-1 viral p24 capsid in the cell culture supernatants at day 3 post-infection. Comparable antiviral activities were found between monomeric and trimeric C34 peptides, which both inhibited 90% of HIV-1VN44 and HIV-1Bal replication when used at 10^−6^ and 10^−7^ M (Figure 6A,B). However, in this assay, similar antiviral activities were found between monomeric and trimeric N36 (Figure 6). Because this assay takes place over 3 days with only one treatment with monomeric or trimeric peptides at the initial day of the infection and before the addition of the viral inoculum in the cell culture, we wondered whether the stability of monomeric and trimeric peptides was affected after several days in a serum-rich culture medium.

### Stability of C34 and N36 monomeric and trimeric peptides in physiological-like medium

**Figure 7:**
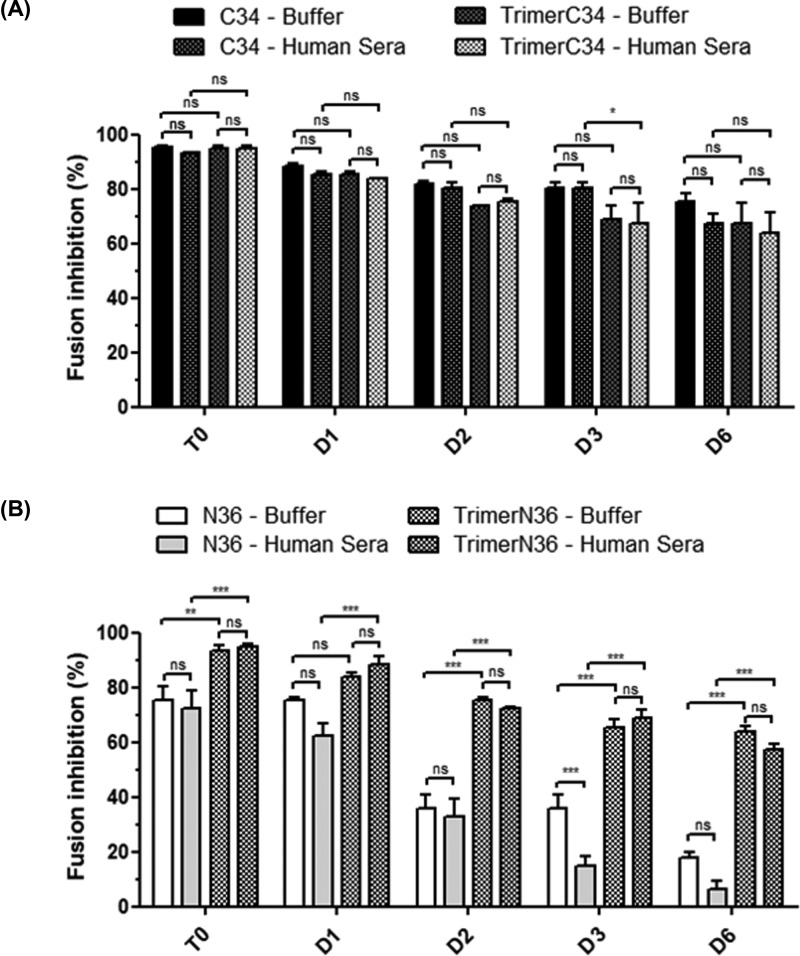
Stability of monomeric and trimeric C34 and N36 in physiological medium Stability of monomeric and trimeric C34 (**A**) and N36 (**B**) in physiological medium containing 50% of human AB serum. Each peptide (5 × 10^−7^ M) was incubated for 1–6 days in this medium. In parallel, each peptide was also incubated in cell culture medium alone. The stability of each peptide was evaluated by assessing the conservation of its antiviral activity to inhibit syncitia formation. *, *P*<0.005; **, *P*<0.001; ***, *P*<0.0001; ns, non-significant.

To test the stability of monomeric and trimeric C34 and N36 peptides, each peptide was incubated for 0, 1, 2, 3 or 6 days in PBS buffer or cell-culture media complemented with 50% of human AB serum, before testing its anti-fusion activities in the inhibition of syncytia formation. Our findings demonstrated that both monomeric and trimeric C34 peptides at 5 × 10^−7^ M inhibited 80–90% of syncytia formation either in PBS or in the medium with 50% AB serum. Similar anti-fusion activity was measured between the monomeric and trimeric forms of the C34 peptides when tested at day 0. However, the anti-fusion activity of C34 peptides gradually decreased for both the monomeric and trimeric forms from days 1 to 6, reaching 30–40% of anti-fusion activity at day 6. No significant differences depending on the time factor were noted between monomeric and trimeric C34 peptides, whether in PBS or in the medium with 50% AB serum (Figure 7). In parallel, the monomeric N36 peptide at 5 × 10^−7^ M inhibited 70% of syncytia formation while the trimeric form was more potent and inhibited 90% of syncytia formation in either PBS or serum-containing medium when tested at day 0. Remarkably, when the anti-fusion activities of monomeric and trimeric N36 peptides were tested under the same conditions as noted above, a gradual loss of the antiviral activity was observed over time, reaching 85 and 60% of anti-fusion activity, respectively, after 6 days of pre-incubation. Interestingly, no additional loss of antiviral activities of monomeric and trimeric N36 and C34 peptides was obtained after their preincubation in cell culture media complemented with 50% human AB serum compared with incubation in PBS alone. Collectively, our results show that the trimerization of C34 peptide seems to have no beneficial effect on its antiviral activity or its stability, while the trimerization of N36 peptide seems to strengthen both its anti-fusion activity and its stability.

## Discussion

Inhibitors of HIV-1 infection targeting the crucial step of viral entry can be divided into three groups: (***i***) inhibitors of the interaction between HIV-1 glycoprotein and its receptors CD4 and its co-receptors CCR5/CXCR4, (***ii***) inhibitors of the maturation of HIV-1 envelope glycoprotein gp160 precursor and (***iii***) fusion inhibitors. The most renowned of the first class of inhibitors, approved by the FDA is Maraviroc, which acts as a CCR5 antagonist by preventing gp120–CCR5 interaction. Despite potent antiviral activity, its delivery for therapeutic use must be managed with caution and it must be administered to patients carrying only HIV-1 R5 tropic virus. Otherwise, such treatment would promote the rapid emergence of X4 and X4/R5 tropic viruses, and lead to a rapid evolution of the disease to AIDS [20].

The second class of inhibitor targets furin/PC endoprotease, a crucial cellular enzyme responsible for the maturation of the precursor envelope glycoprotein of HIV-1 gp160 into gp120 and gp41 at a specific dibasic endo-cleavage site. Elimination of this site by site-directed mutagenesis leads to unprocessed gp160, which conserves its high affinity with CD4 receptor [36] but generates virologically inactive particles despite the fact that they are morphologically similar to the wild-type (WT) particles, as shown by electron microscopy [37]. Controlling cellular proteases for efficient viral maturation is a common feature of a broad range of enveloped viruses including retroviruses, influenza [12], measles [14], Ebola [13], bovine leukemia [38] and Borna disease viruses [39]. Thus, blocking this key step of the viral envelope maturation has considerable importance for therapeutic applications that can be achieved by the development of peptidic or non-peptidic inhibitors targeting the involved host endoproteases [40,41]. One striking advantage of this strategy is that host targets are not subjected to continuous variability as is the case for RNA viruses. This high genome variability is the main mechanism by which viruses escape control by the immune system and antiviral drug treatments.

The third class of inhibitors is the anti-fusion peptides that act essentially by blocking the fusion between the viral and host cell membranes. Several molecules derived from HIV-1 gp41 glycoprotein have been evaluated or are under investigation, including peptide inhibitor analogs of HRI and HRII such as N36 and C34 peptides [42–44]. T20 peptide, the only peptide entry inhibitor that has been approved by the FDA, is a 36 amino acid peptide derived from the HRII domain of gp41. Unfortunately, its administration in therapeutics has been reduced over the years due to several limitations including (***i***) its low half-life of approximately 4 h in plasma, which requires two injections per day, (***ii***) its low solubility and (***iii***) the selection of emerging T20-resistant viruses [45]. Therefore, further studies have focused on modifying the T20, C34 and N36 peptides and their various analogs to produce new HIV-1 entry inhibitors with better antiviral activities and fewer limitations. Accordingly, the addition of an artificial tail anchor to the C-terminus of C34 analogs enhanced by approximately 77-fold of its antiviral activity [46]. Similarly, the addition of two M-T hook residues to the SC29 EK peptide or its COOH linking to C16-fatty acid chain to its short analog HP23 peptide, named LP11, greatly improve their antiviral acivities but also with a significant enhancement of the stability of the HP23-lipopeptide [47]. More recently, an inovative approche based on the association of the D1D2 domains of CD4 and a fusion inhibitory peptide has been reported [48,49]. This new generation of HIV-1 inhibitors are characterized by their capacity to act at two levels, first, by inactivating, free viral particles following their association via D1D2 domains of CD4, and second, by blocking the mechanism of viral and cell host membrane fusion. In a parallel approach, the mutation in CHR of the non-essential amino acid residues in CHR–NHR interactions, allowed to be enhanced by approximately 26-fold the antiviral activity of the mutated peptide [50]. In our study, N36 and C34 trimeric peptides were obtained by using a minimal linker, composed of only five lysine residues to enhance the solubility and four O_2_Oc groups as flexibility spacers at branching points. Each trimeric peptide was synthesized, using the Fmoc-amino-acid approach, on a free tail of the linker and purified to homogeneity [32,33]. The advantage of this approach is to mimic the trimeric conformation of the NHRI and CHRII domains present on the transmembrane envelope of HIV-1 gp41 viral particles. Their physicochemical analysis by circular dichroism of monomeric and trimeric N36 and C34 peptides demonstrated: (***i***) weak α helical propensity structures in monomeric N36 peptides and their absence in monomeric C34 peptides, (***ii***) increased rate of α helical structures in trimeric N36 and absence of helicity structures in trimeric C34, (***iii***) presence of great α helical structures in the mixture of (monomeric N36 + trimeric C34) and in that of (monomeric C34 + trimeric N36) indicating, on one hand the capacity of monomeric peptides to interact with their heterologous trimeric peptides and, on the other hand, the capacity to form six α helix bundles. These results indicate that our strategy for the design of NHRI and CHRII trimeric regions of HIV-1 gp41 is compatible with their abilities to interact with their complementary regions and to form α helical structures. Our group [22,23] has previously demonstrated that such structures can also be formed, with rapid kinetics and in a stable structure compatible with six α structure bundles, when monomeric N36 and monomeric C34 are mixed together in an equimolar stoichiometry.

The antiviral activities of monomeric and trimeric NHRI and CHRII peptides were tested for their abilities to block both cell–cell fusion in a HeLa-cell-based assay and viral replication in single or multiple viral cycle replication assays. Our results with monomeric N36 and C34 peptides are in agreement with data reported by other groups [51–54], and show high antiviral activity of the monomeric C34 with an IC_50_ in the nanomolar range. In contrast, only a modest antiviral activity was obtained with monomeric N36, with IC_50_ in the μM range. At a comparable level, no significant beneficial effect was observed following the trimerization of C34 peptide, which conserved antiviral activities similar to those of its monomeric counterpart. Interestingly, the trimerization of N36 peptides strongly enhanced its antiviral activities: by 100-times against R5-tropic viruses and by 500-times against X4-tropic viruses. These results are in accordance with those reported by Chen et al. [54], but are in apparent contradiction with those of Nakahara et al. [55] and Nomura et al. [35], which reported only a modest antiviral activity of their trimeric N36 peptide. This discrepancy may be related to the fact that their N36 peptide was coupled to the template via its N-terminal region while, in our study, trimeric N36 peptide was assembled via its C-terminal region, an orientation that seems to mimic the flexibility of this region on the structure of HIV-1 gp41 in a better way. Our findings regarding the antiviral activity of monomeric C34 peptide also differ from those of the same group where they reported only a relatively weak antiviral activity, 100-times lower than that found in the present study. This difference could be explained by the two additional amino acids of arginine and glutamic acid at the C-terminal of C34 peptide (C34 RE). However, trimeric C-peptides and N-peptides, produced by various strategies, have been described as efficient HIV-1 fusion inhibitors [51,56,57]. These studies show that trimeric peptides coupled to various carriers, known for their capacity to induce coiled-coil trimerization with α helix structures, retain at least the same antiviral potency as the monomer [57–60]. However, one obstacle of these approaches is related to the immunogenicity of the relatively long amino-acid sequences of the carriers/linkers such as foldon, the natural trimerization domain of T4 bacteriophage fibritin (27 amino-acids) [54] or the coiled-coil trimerization GCN4 motif (34 amino acids) [61]. In addition to the issue related to their immunogenicity these linkers/carriers are not easy to synthesize chemically. Although this limitation can be circumvented by engineering recombinant fusion polypeptides [58] or by using smaller linkers, this approach often requires several difficult steps in order to couple the peptides to the linker [62].

The C34 peptide, derived from the HR2 domain, is a 34 amino acid peptide which exhibits a potent antiviral activity at the nanoMolar range [22,23]. However, this C34 peptide cannot be used in human therapy because of its low half-life and low-solubility. Similar limitations were reported with the N36 peptide, an analog of the HRI domain that presents a low solubility as well as weak antiviral activities (μM) [22,23]. To circumvent these limitations, novel HRI and HRII peptide analogs such as Sifuvirtide, SC34EK and C34M2 have been developed [42–44]. These peptides present improved activities compared with T20, N36 or C34 and are active against T20-resistant HIV-1 stain. Nevertheless, the solubility and stability of such peptides remain to be further characterized [42–44].

A major point for the development of antiviral peptides for use in human therapy is related to their bioavailability and stability. Once delivered into the physiological medium, peptides are exposed to protease activities and transporters that may be deleterious for these properties. In the present study, we tested the conservation of the antiviral activities of monomeric and trimeric N36 and C34 peptides. The peptides were incubated in a cell culture medium complemented with 50% of human serum AB or in the PBS buffer as a control. The antiviral activities of 5 × 10^−7^ M of each peptide were tested at days 0, 1, 2, 3 and 6 post-incubation. Our findings demonstrated stable and similar antiviral activities for monomeric and trimeric C34 peptides. This stability was also observed with the trimeric N36 peptide but not with the monomeric N36 peptide, which had lost more than 75% of its antiviral activity at days 3 and 6. These data underline the important role of the trimerization of N36 peptide, not only to increase its antiviral activity but also to strengthen its stability in a medium containing human serum used to mimic the *in vivo* physiological medium.

The design of the trimeric or hexameric forms of the NHR and CHR domains of HIV-1 and also of a very large number of enveloped viruses could be considered as an innovative alternative for the development of such peptide inhibitors intended to block or reduce the viral load in the case of acute infections with the most highly pathogenic viruses such as Ebola or influenza. Such momentary, targeted treatment may allow a window of time to be established, during which the immune response takes place to mount an efficient antiviral response. This type of peptide design could also be considered as an approach for the development of synthetic or recombinant tools to be used as microbicides or immunogens for the development of novel candidate vaccines with predetermined specificities. Our findings presented in the present study, in agreement with those reported by other groups [42,52,63–65], can be considered as a proof of concept validation to continue the development of various trimeric analogs of NHRI and CHRII domains of HIV-1 gp41 endowed with greater antiviral activities and having better stability and bioavailability. It seems essential to extend this approach to other, shorter peptide or lipopeptide analogs of T20, C34 and N36 reported recently in the literature [42,52,63–65].

## Abbreviations

AIDS: acquired immune deficiency syndrome
CHRII: C-terminal heptad region
HAART: highly active antiretroviral therapy
HIV-1: human immunodeficiency virus 1
NHRI: N-terminal heptad repeat
O_2_Oc: 3.6 dioxaoctanoic acid
